# Vitiligo and Prostate Cancer Correlation

**DOI:** 10.7759/cureus.59349

**Published:** 2024-04-30

**Authors:** Richard L Siwicki, Jeremy Shore, Robert A Norman

**Affiliations:** 1 Biomedical Sciences, University of South Florida, Tampa, USA; 2 Medical School, Nova Southeastern University Dr. Kiran C. Patel College of Osteopathic Medicine, Fort Lauderdale, USA; 3 Dermatology, Nova Southeastern University Dr. Kiran C. Patel College of Osteopathic Medicine, Fort Lauderdale, USA

**Keywords:** psa, vitamin d, biopsy, prostatic adenocarcinoma, prostate cancer, vitiligo

## Abstract

A 72-year-old male with a history of systemic hypertension, asthma, chronic obstructive pulmonary disease (COPD), and hyperlipidemia presents with diffuse patches of cutaneous depigmentation. A shave biopsy of different regions of depigmented skin indicated vitiligo. The patient was prescribed Opzelura (ruxolitinib) 1.5% topical cream as well as tacrolimus 0.1% topical ointment for vitiligo. He also had a history of prostate cancer. A prostate biopsy revealed three sites of prostatic adenocarcinoma with a Gleason score of 6 and a Prostate Imaging-Reporting and Data System (PI-RADS) score of 2. The patient remained in active surveillance for prostate cancer without treatment, due to its low severity. A subsequent biopsy five years later revealed a decrease in prostate cancer prevalence, with cancer present in only one core and at a lower severity. The purpose of this case presentation is to discuss possible links between vitiligo and prostate cancer, as well as their shared mechanisms and pathways.

## Introduction

Vitiligo is an autoimmune disorder that results in the depigmentation of the skin due to the destruction of pigment-producing melanocytes. These white lesions usually appear somewhat symmetrically on both sides of the body and usually first appear at the digits, arms, and face. While the most obvious symptom is cosmetic, the effects of this condition can stretch far beyond, often manifesting in poor mental health and worsening self-image [1].

Vitiligo, like other autoimmune disorders, is associated with various comorbidities, including thyroid disease, Addison's disease, and lupus [2]. A less-explored comorbidity, however, is prostate cancer. Though the association between prostate cancer and vitiligo is unclear, some studies have shown a correlation between the presence of the two conditions [3,4], as well as an overall increase in the prevalence of all malignancies in people suffering from vitiligo [3]. Hospital-based studies show that vitiligo is present in 2.5% of people in African hospitals, 1.6% of people in Asian hospitals, and 1.5% of people in American hospitals. Around 1.1% of males and 1.3% of females have vitiligo [5]. Although the pathophysiology of any potential association between vitiligo and prostate cancer is largely unknown, there are mechanisms and trends shared between the two conditions that may hold the key to this phenomenon.

## Case presentation

**Figure 1:**
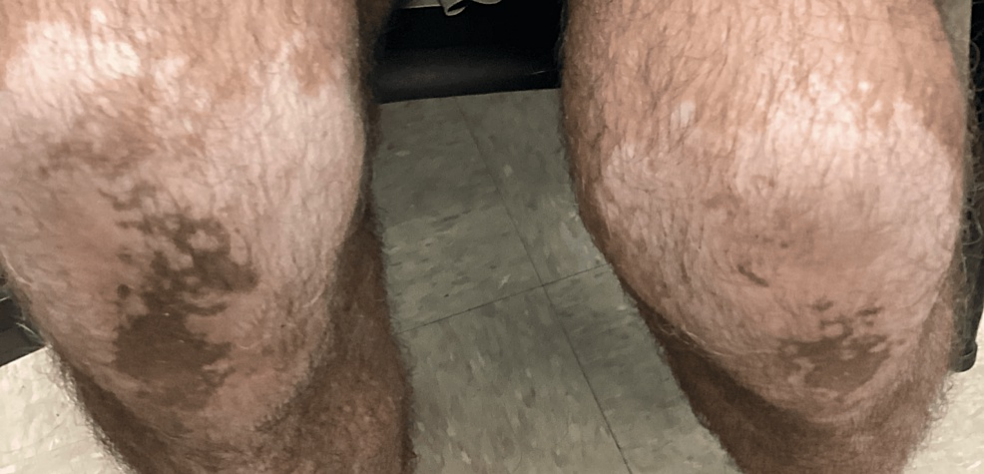
Knees and shins of the patient Depigmentation of knees and shins, bilaterally

**Figure 2:**
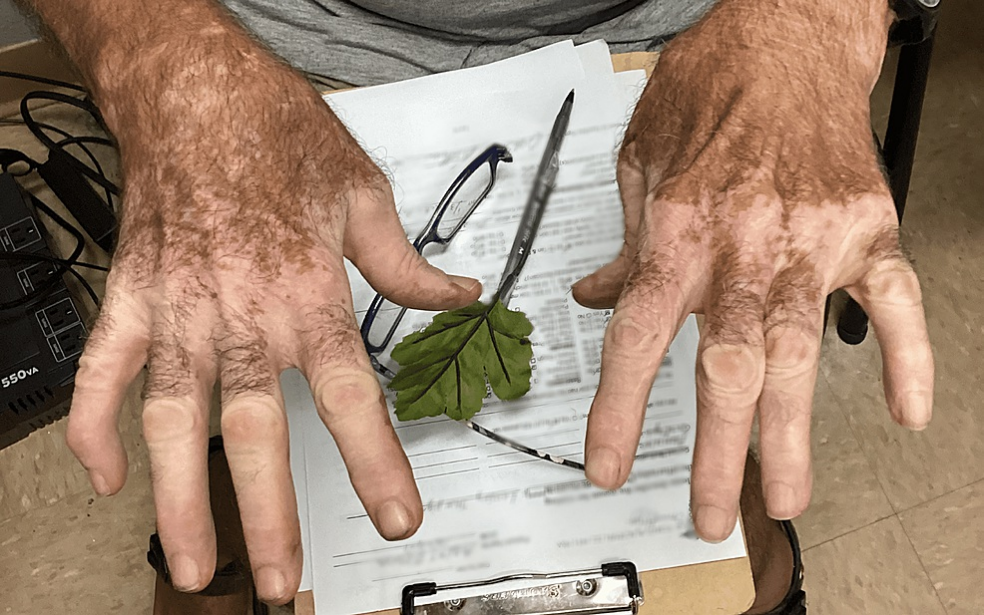
Hands of the patient Vitiligo-associated depigmentation of the digits

A 72-year-old white male with a history of systemic hypertension, asthma, chronic obstructive pulmonary disease (COPD), and hyperlipidemia presented to the office with patchy depigmented lesions on the knees, shins, toes, hands, fingers, and elbows (Figure 1 and Figure 2). The patient explained that he noticed these lesions approximately eight years ago and that the depigmentation had recently become more prominent. He claims no history of thyroid disease. The patient is a frequent smoker, smoking 1-2 cigars per day over the past 40 years.

**Figure 3:**
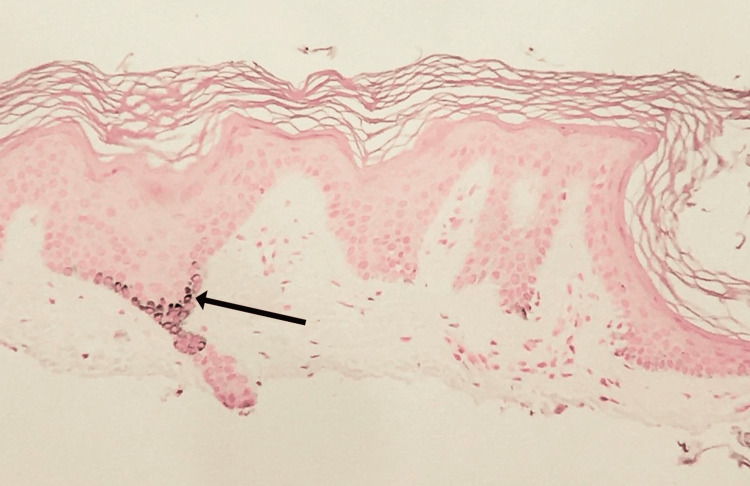
Fontana-Masson stain of the biopsied tissue Stain reveals incomplete loss of basilar melanin pigmentation. The area specified by an arrow (darkly stained tissue) indicates a small section containing melanin

**Figure 4:**
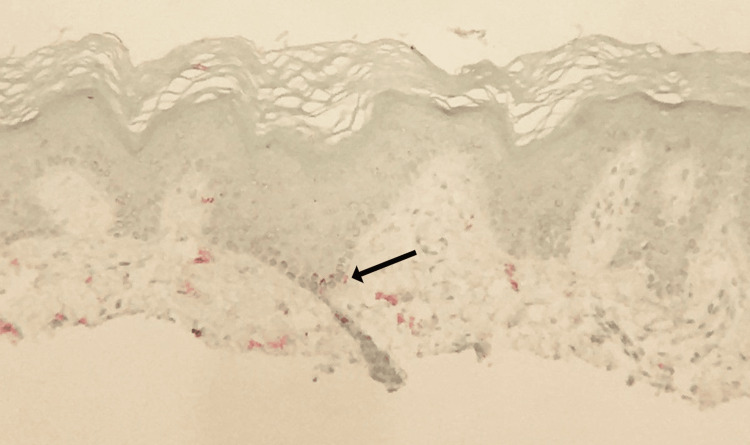
Mart-1 immunostain of the biopsied tissue Stain reveals loss of melanocytes in the basal layer. The area specified by an arrow (red stain) indicates a small section with melanocyte presence. The red stain is virtually absent from the remainder of the tissue sample

To address the depigmentation, shave biopsies (Figure 3 and Figure 4) were performed on the right knee and left elbow to differentially rule out vitiligo. The right knee specimen was found to have little inflammatory infiltrate and mild hyperkeratosis, with a high loss of junctional melanocytes. The area also showed inconsistent pigmentation within the depigmented area. The left elbow specimen was also found to have little inflammatory infiltrate and mild hyperkeratosis, with less, but still significant, loss of junctional melanocytes. Both biopsies showed results consistent with vitiligo and came back negative for fungal organisms, leading to a diagnosis of vitiligo.

In 2018, the patient presented to his urologist with lower abdominal pain. He subsequently underwent a transrectal ultrasound-guided 12-core prostate biopsy which returned positive for prostate cancer on three of the 12 cores. Prostatic adenocarcinoma was identified on the left lateral mid, right base, and right lateral mid areas of the prostate, with the tumor involving 30%, <5%, and 10% of the biopsy length, respectively. The Gleason score for each positive core on both 12-core prostate biopsy procedures was 6, and the Prostate Imaging-Reporting and Data System (PI-RADS) score was 2, indicating low metastatic risk and slow growth [6] and low likelihood of developing clinically significant cancer [7]. The patient maintained active surveillance due to the low relative risk and periodically visited his urologist between 2018 and 2023, with frequent testing of prostate-specific antigen (PSA) levels. Over the years, the patient's serum PSA increased from 10.4 ng/mL in May 2018 to 13.57 ng/mL in August 2023, raising some concerns. In late 2023, a few days after he presented with worsened vitiligo symptoms, the patient received another 12-core prostate biopsy which returned positive for prostate cancer on only one of the 12 cores, at the left mid area. The positive core also had a Gleason score of 6 as well as a PI-RADS score of 2, similar to that of the cores from the first biopsy. However, the tumor only involved 5% of the biopsy length, indicating lower severity.

## Discussion

The immune-mediated nature of melanocyte destruction in vitiligo naturally results in increased coincidence with other autoimmune diseases, including Hashimoto's disease, lupus, and Addison's disease [2]. Prostate cancer, however, is a lesser-known comorbidity with vitiligo. In a 2018 study, males with vitiligo were shown with statistical significance to be 3.1 times more likely to coincidentally suffer from prostate cancer than the average male population without vitiligo, as well as generally having a higher incidence of all malignancies (0.71 per 100 person-years as compared to 0.28 in the general public) [3]. On the contrary, a study in 2021 with data from the National Health Insurance Research Database of Taiwan refutes the idea that overall malignancies are increased in vitiligo patients, finding cancer rates of 621.06 per 100000 person-years (or 0.62 per 100 person-years) among vitiligo patients compared to 726.99 (or 0.73) across the general population. However, this study still found a higher incidence of prostate cancer in these vitiligo patients, although analysis proved it to be insignificant [4].

A potential component of the physiological relationship between vitiligo and prostate cancer includes various cytokines associated with vitamin D_3_. In patients with vitiligo, as with many other autoimmune disorders, serum levels of vitamin D_3_ and its analogs are frequently low compared to control groups. One study showed that as many as 68.9% of vitiligo patients were deficient in varying degrees in 25-hydroxyvitamin D_3_ (calcifediol) [8]. Although a causal relationship was not found between vitiligo and vitamin D deficiency [9-11], vitamin D is shown to promote melanogenesis, whereas vitiligo entails an autoimmune response against melanocytes, leading to decreased areas of melanin [10]. Vitamin D_3_ and other forms of vitamin D have also been heavily investigated as far as their influence in the prevention and treatment of prostate cancer. Prostate cells are known to contain vitamin D receptors, which bind some vitamin D analogs, predominantly 1,25-dihydroxyvitamin D_3_ (calcitriol). This pathway results in the transcription of genes encoding CDK inhibitors, which curtail rapid cell division [12]. As such, higher serum vitamin D concentration may possibly hinder the proliferation of prostate tumors.

Furthermore, an increased dietary calcium intake was associated with increased prostate cancer risk, potentially due to its lowering effect on 1,25-dihydroxyvitamin D_3_ levels [13]. Information regarding inherent serum vitamin D concentration, from sunlight or otherwise, is similarly inconclusive but provides more evidence supporting its properties as an antitumor agent. Many studies claim that increased endogenous levels of 1,25-dihydroxyvitamin D_3_ and 25-hydroxyvitamin D_3_, which is converted to 1,25-dihydroxyvitamin D_3_ by various organ tissues (including the prostate), are correlated with lower incidence and/or aggressiveness of prostate cancer. One study demonstrated that high serum vitamin D levels (≥80 nmol/L) were correlated with a higher incidence of prostate cancer, due to the overexpression of 24-hydroxylase, a vitamin D-deactivating enzyme [14].

While the studied effect of vitamin D on the incidence of prostate cancer is conflicting, information regarding its effect on the management and progression of prostate cancer is less ambiguous. One study demonstrated that higher serum vitamin D levels were correlated with lower prostate cancer aggression, at a rate of a 9% decrease in mortality per 20 nmol/L increase in 25-hydroxyvitamin D_3_ concentration [9]. In addition, vitamin D supplements have been shown to reduce PSA concentration and thus likely slow the progression of prostate tumors [15].

Another important factor in the relationship between vitiligo and prostate cancer is the mechanism of interferon-gamma (IFN-γ). IFN-γ is thought to be the driving force behind the onset and progression of vitiligo. Through the same JAK-STAT pathway with which it mediates the immune response, IFN-γ activates the transcription factor STAT1. STAT1 promotes the transcription of genes that encode CXCL9 and CXCL10, chemokines which recruit CD8+ cytotoxic T cells. These cells migrate to the skin and other melanocyte-dense regions and cause melanocyte apoptosis, which manifests in the depigmented lesions seen in vitiligo [16].

The elevated level of IFN-γ in many vitiligo patients has many implications when considering its association with prostate cancer. IFN-γ is thought to have both pro- and anti-tumor effects on prostate cells. TRAMP cells, cancerous epithelial cells found in the prostates of mice, are notable for their similarity to human prostate cancer. Apoptosis of TRAMP cells has been found to be regulated by IFN-γ [17]. Interestingly, the same mechanism also appears to have metastatic effects on prostate cancer cells. IFN-γ signaling can cause epithelial-to-mesenchymal transition in prostate cancer cells, a process by which cells lose tethering features, including cell junctions and polarity, and become mobile [18]. This process allows cancer cells to detach from a tumor and causes metastasis. As such, any association between vitiligo and prostate cancer is more likely to originate from reduced vitamin D as opposed to increased IFN-γ levels, as IFN-γ has many offsetting effects on prostate cancer proliferation. 

The serum vitamin D levels of the patient either were not measured or were unavailable. However, before both of the 12-core prostate biopsies, his PSA levels were measured and determined to be abnormally high. At the time that the patient complained about increased prominence of vitiligo, his serum PSA levels had overall increased since his first prostate biopsy, with values being much higher than the generally accepted normal threshold of 4.0 ng/mL [19]. As confirmed by the biopsies, these numbers indicate a significant chance of prostate cancer presence. PSA, as previously explored, has been linked to vitamin D insofar as supplements containing vitamin D can reduce its serum concentration [15]. As such, it is entirely possible that the patient's high PSA levels are related to low serum vitamin D, although this has not been confirmed through blood testing. 

While this information is not nearly sufficient to show an inherent link between vitiligo and prostate cancer in this patient, it does establish a potential cause for comorbidity through the mechanism of vitamin D deficiency. The patient was prescribed Opzelura (ruxolitinib) 1.5% topical cream, an inhibitor of the JAK1 and JAK2 proteins prevalent in the immune reaction responsible for vitiligo [20], as well as tacrolimus 0.1% topical ointment, another immunosuppressant, and was instructed to apply them over the affected area. 

Due to the low relative risk of prostate cancer metastasis as indicated by the Gleason score of the positive biopsy cores, the patient was put on an active surveillance program for his prostate cancer, with no direct treatment contingent on stable serum PSA levels. The patient had trouble urinating with a smaller but still stable stream, but both the patient and the physician agreed to hold off on pharmacotherapy for the dysuria.

## Conclusions

Here, a case was presented of a patient with vitiligo in addition to prostate cancer to share clinical metrics related to the disease state, allowing one to better understand a potential connection between the presence of prostate cancer and vitiligo. Although any mechanism that relates the presence of vitiligo with prostate cancer remains unclear in current research, much research links the deficiency of vitamin D or altered expression of vitamin D receptors on cells with increased rates of cancer in people. Additionally, with a documented trend for patients with vitiligo to be vitamin D deficient and instances of cancer being related to vitamin D deficiency in non-vitiligo patients, a mechanism that relates vitamin D deficiency with a webwork of altered immunological events to produce vitiligo and prostate cancer is speculated to be a likely explanation for the presence of vitiligo and prostate cancer in this patient. More research is needed specifically to determine how vitamin D deficiency leads to the increased expression of pro-inflammatory cytokines (leading to specific autoimmune diseases) in addition to how vitamin D deficiency leads to altered biochemical events in specific cell types, leading them to become cancerous. Causal biochemical research is needed specifically to study the relationship between autoimmunity processes and related processes that are also present in or interface with the presence of cancer.
